# Step Detection and Activity Recognition Accuracy of Seven Physical Activity Monitors

**DOI:** 10.1371/journal.pone.0118723

**Published:** 2015-03-19

**Authors:** Fabio A. Storm, Ben W. Heller, Claudia Mazzà

**Affiliations:** 1 Department of Mechanical Engineering, University of Sheffield, Sheffield, United Kingdom; 2 INSIGNEO Institute for *in silico* Medicine, University of Sheffield, Sheffield, United Kingdom; 3 Centre for Sports Engineering Research, Sheffield Hallam University, Sheffield, United Kingdom; University of Manchester, UNITED KINGDOM

## Abstract

The aim of this study was to compare the seven following commercially available activity monitors in terms of step count detection accuracy: Movemonitor (Mc Roberts), Up (Jawbone), One (Fitbit), ActivPAL (PAL Technologies Ltd.), Nike+ Fuelband (Nike Inc.), Tractivity (Kineteks Corp.) and Sensewear Armband Mini (Bodymedia). Sixteen healthy adults consented to take part in the study. The experimental protocol included walking along an indoor straight walkway, descending and ascending 24 steps, free outdoor walking and free indoor walking. These tasks were repeated at three self-selected walking speeds. Angular velocity signals collected at both shanks using two wireless inertial measurement units (OPAL, ADPM Inc) were used as a reference for the step count, computed using previously validated algorithms. Step detection accuracy was assessed using the mean absolute percentage error computed for each sensor. The Movemonitor and the ActivPAL were also tested within a nine-minute activity recognition protocol, during which the participants performed a set of complex tasks. Posture classifications were obtained from the two monitors and expressed as a percentage of the total task duration.

The Movemonitor, One, ActivPAL, Nike+ Fuelband and Sensewear Armband Mini underestimated the number of steps in all the observed walking speeds, whereas the Tractivity significantly overestimated step count. The Movemonitor was the best performing sensor, with an error lower than 2% at all speeds and the smallest error obtained in the outdoor walking. The activity recognition protocol showed that the Movemonitor performed best in the walking recognition, but had difficulty in discriminating between standing and sitting. Results of this study can be used to inform choice of a monitor for specific applications.

## Introduction

An objective and reliable method for the classification and quantification of free-living motor activity is a prerequisite for the understanding of the complex relationship between health and physical activity. Recently, the use of motion sensors for its estimation has gained widespread recognition. Accelerometry is currently the most exploited technology in physical activity monitoring [1]. The development of micro-engineered piezoresistive and capacitive accelerometers, often referred to as microelectromechanical systems (MEMS), allows the recognition of both dynamic and static activities [2]. These devices have been additionally combined with physiological sensors such as heart rate, temperature, heat flux and galvanic skin response with the aim of increasing their accuracy of predicting energy expenditure and discriminating activity types [3–5]. The integration of these technologies in a sensor fusion approach has been investigated in the last decades [6].

Various commercially available physical activity monitors (PAMs) have been tested and validated for field-based research in both healthy and chronically diseased populations [7]. Most recent studies in this field investigated the validity of energy consumption algorithms embedded in different devices [8–10]. A review by Welk et al. [11], focusing on protocol equivalency, emphasized the “emerging measurement challenge” caused by the increasing availability of low cost PAMs, along with the chronic difficulty in comparison and standardization of data from different models of accelerometry-based sensors. As far as is known to the authors, only one study has investigated consumer-based PAMs, i.e. PAMs addressed directly to final users, typically interested in health and fitness rather than to clinicians or therapists. The devices investigated in this study showed an absolute error for energy expenditure estimation during a 69-minute protocol ranging between 9.3% and 23.5% [12].

Activity type-specific equations are generally implemented into PAMs to model energy expenditure [13]. Interestingly, despite the fact that in these devices the application of these activity-dependent equations relies on step detection, only a few studies have focused specifically on the accuracy of this estimate. Dijkstra et al. [14] compared the accuracy of a pedometer (Yamax Digi-walker SW 200) and a triaxial accelerometer (Dynaport Micromod) in their step count estimates in patients with Parkinson’s disease, using video recordings as reference. The error of the pedometer was speed-dependent, ranging between 4.5%- 17.2%, whereas the error of the accelerometer was around 7%. A second study [15], testing and comparing a pedometer (Digiwalker SW701) and a multisensor device (Sensewear Armband Mini) in patients with chronic obstructive pulmonary disease and healthy elderly showed that at slow speeds (1.6 ± 0.2 km/h) neither of the two systems was adequately accurate.

The robustness of the measure during walking at slow speed is of particular interest in clinical research [16]. An additional factor that could affect the accuracy of step detection is, of course, the walking environment. To our knowledge, however, the accuracy of step count in PAMs has never been compared between indoor and outdoor settings.

The aim of this study is to compare the step count detection accuracy of seven different PAMs in healthy adults, covering a range of technologies and prices, during different walking protocols, including indoor and outdoor walking at different speeds. Among these monitors, those that allow recognition of common everyday tasks will be further tested in their ability to discriminate and classify basic activities within more composite motor tasks. The results of this study may be used to provide reference values for the error to be expected when the investigated PAMs are used for long-term recording of physical activity.

## Materials and Methods

### Participants

Sixteen participants were recruited for the study. The sample characteristics are shown in Table 1. Participants did not report any impairment or morbidity that could interfere with the assessment of physical activity.

**Table 1:** Sample Characteristics of the study group (mean ± SD).

Characteristics	Value
**Men/Women**	10/6
**Age (y)**	28.87 ± 2.65
**Weight (kg)**	72.0 ± 9.2
**Height (m)**	1.75 ± 0.09
**BMI (kg/m^2^**)	23.5 ± 2.3

### Ethics statement

Approval from the University of Sheffield Research Ethics Committee was obtained for the study and participants were asked to read carefully an information sheet before giving written informed consent.

### Physical Activity Monitors

**Fig 1:**
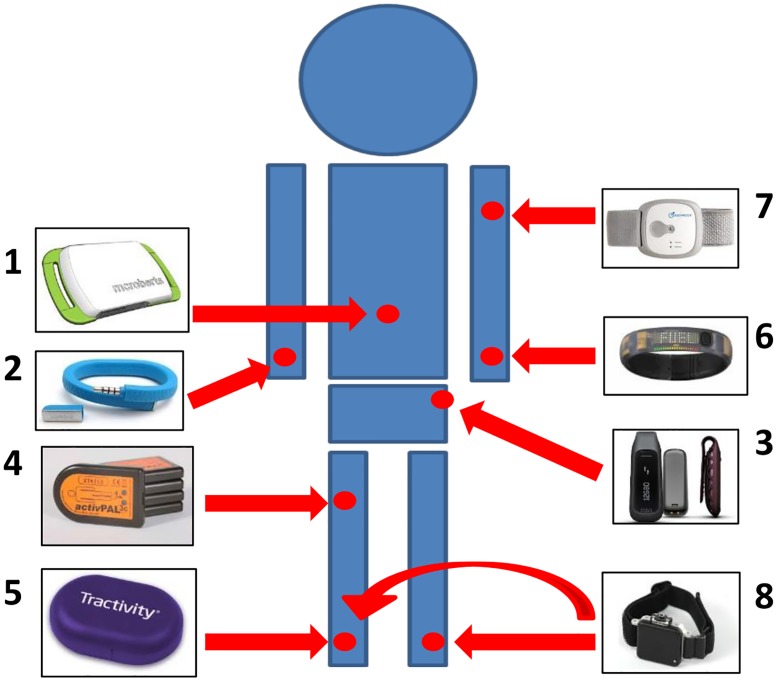
Sensor placement. The figure shows the location of the sensors on a subject’s body: Movemonitor (1), Up (2), One (3), ActivPAL (4) Tractivity (5), Nike+ Fuelband (6), Sensewear Armband Mini (7), OPAL (8).

Seven different PAMs were assessed during this study: Movemonitor (Mc Roberts, The Hague, The Netherlands), Up (Jawbone, San Francisco, USA), One (Fitbit, San Francisco, USA), ActivPAL (PAL Technologies Ltd., Glasgow, UK), Nike+ Fuelband (Nike Inc., Beaverton, USA) Tractivity (Kineteks Corp., Vancouver, Canada) and Sensewear Armband Mini (Bodymedia, Pittsburgh, USA). Further details about these sensors are provided in Table 2. After having their anthropometric characteristics recorded, the subjects were fitted with the sensors, which were all positioned at the manufacturer’s recommended locations (Fig. 1). The participants were asked to perform two protocols, one including different locomotion tasks and one including different postural transitions and complex motor activities.

**Table 2:** Details of the monitors tested in this study.

Instrument	Sensor Type	Location	Outputs	Output Data Aggregation	Data Interface and version	Price
**DynaPort Movemonitor (Mc Roberts)**	Triaxial Accelerometer	Lower back	Time sitting, lying, standing, locomotion, shuffling, steps	1s epochs	*Dyrector Ver*. *1*.*0*.*7*.*17–*Web based data server	800 €
**UP (Jawbone)**	Triaxial Accelerometer	Wrist (right)	Steps	60s epochs data and graphics by day or min	*UP Ver*. *2*.*8*.*8*.*3*.*7*.*1—*App	114 €
**One (Fitbit)**	Triaxial Accelerometer	Waist (left)	Steps	60s epochs data and graphics by day or 15 min aggregation	*Connect Ver*. *1*.*0*.*0*.*4022—*Web based software	106 €
**ActivPAL (PAL Technologies)**	Triaxial Accelerometer	Shank (right)	Time sitting and lying, standing, stepping, steps	1s epochs	*ActivPAL Ver*. *7*.*1*.*18—*PC based software	1,277 €
**Tractivity (Kineteks Corporations)**	Uniaxial Accelerometer	Ankle (right)	Steps	60s epochs data and graphics by day or hour	*Connect Ver*. *2*.*12—*Web based software	18 €
**Nike+ Fuelband (Nike)**	Triaxial Accelerometer	Wrist (left)	Steps	60s epochs data and graphics by day or hour	*Nike+ Connect Ver*. *3*.*8—*Web based software	171 €
**Sensewear Armband Mini (Bodymedia)**	Triaxial Accelerometer, Heat Flux, Galvanic Skin Resp., Skin Temp.	Upper left arm at triceps	Steps	60s epochs data and graphics by day or hours or minutes	Sensewear Ver. 7.0.0.2378—PC based software	2,400 €

### Experimental protocol

**Fig 2:**
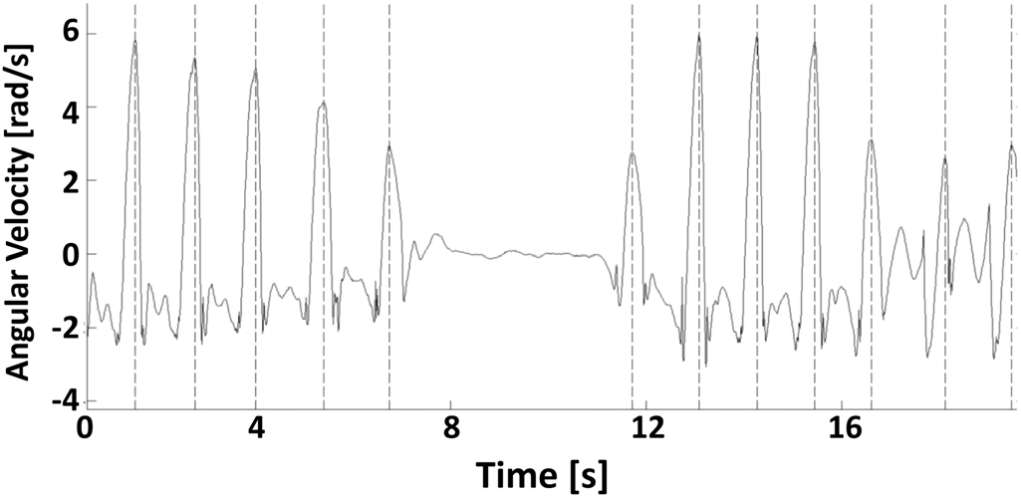
Typical angular velocity signal of the shank in the sagittal plane during consecutive steps. The figure shows the angular velocity signal as measured by one of the shank sensors in the sagittal plane during a portion of a randomly selected indoor walking trial. The shown portion of the signal includes walking, stopping and turning. The maxima detected by the algorithm used to detect single steps are also highlighted (dotted vertical lines).

In addition to the activity monitors, two wireless inertial measurement units (OPAL, ADPM Inc., Portland, OR, USA) were positioned on the left and right shanks, just above the ankle, by means of an elastic strap. Data from the OPAL sensors collecting data at a sampling rate of 128 Hz were used as a gold standard for step detection. An algorithm using the gyroscopic signals was implemented in Matlab R2013a (The Mathworks Inc., USA). This algorithm is directly derived from the one proposed by Aminian et al. [17], which has been extensively validated to detect heel strike and toe off in healthy individuals, and identifies the maxima of the angular velocity around the mediolateral axis of the shank corresponding to the swing phases of the leg from the data (Fig. 2). Not being interested in detecting a specific phase in the gait cycle, we used the maxima instead of the heel strike peak used by Aminian et al. as a conservative solution. Nevertheless, the presence of a heel strike between two subsequent strides was always verified and the independent information from the sensors on the two ankles was used as a cross-check to verify the alternate presence of left and right steps. For each session, step counts for left and right shanks were computed and the total number of steps (N) was obtained by summing up the number of right and left steps.

During the first protocol, which tested the accuracy of the monitors for step detection under different walking conditions, each participant simultaneously wore all the seven monitors. The protocol lasted 11-minutes and included the following tasks: a) walking along a 20-meter long indoor straight walkway; b) descending 24 steps (4 flights of 5,12,3 and 4 steps respectively); c) free outdoor walking; d) ascending 24 steps and e) free walking in an indoor setting. During the free indoor walking the participants were asked to walk inside a 300 m^2^ office space filled with lines of desks and separated by rectilinear corridors, without following any predefined path (they were free to decide which way to go, provided that they would not stop nor make abrupt turns). During the outdoor walking they were instructed to walk along a regularly crowded sidewalk, following a pre-defined route that included straight paths and turns around blocks. This was repeated three times, with the participants being instructed to walk at self-selected natural, slow, and fast speeds. The order of the walking speeds was randomized. A detailed description of the protocol is presented in Table 3.

**Table 3:** Summary of the activities performed during the step detection protocol, their duration and the step count (as obtained by the OPAL sensors) for each walking speed.

Activity Type—Step Detection Protocol	Duration	Slow Speed (N)	Self Selected Speed (N)	Fast Speed (N)
**Indoor walking on a straight walkway**	3 min	**260 ± 42**	**313 ± 44**	**353 ± 37**
**Descending 24 steps**	1 min	**70 ± 11**	**72 ± 7**	**66 ± 11**
**Outdoor walking**	3 min	**330 ± 81**	**378 ± 56**	**460 ± 69**
**Ascending 24 steps**	1 min	**67 ± 6**	**65 ± 7**	**63 ± 7**
**Free indoor walking**	3 min	**267 ± 53**	**309 ± 38**	**350 ± 35**
**TOTAL**	**11 mins**	**986 ± 127**	**1127 ± 103**	**1289 ± 115**

The number of steps, as estimated by each sensor (Ñ), was recorded at the end of each trial and saved for further analysis. An additional analysis was performed in order to investigate differences in step count accuracy between the five different walking phases of the protocol. This phase analysis was performed on the Movemonitor and the ActivPAL data only, since the outputs of the other PAMs do not lend themselves to the extraction of the number of steps in sub-intervals.

During the second protocol, in addition to the two OPAL sensors, the subjects wore the two PAMs (Movemonitor and ActivPAL) that are able to discriminate other activities besides walking. This activity recognition protocol lasted 9 minutes and is described in Table 4. This protocol included motor activities designed to challenge the recognition of basic tasks (e.g. introducing upper body movements while sitting or external accelerations affecting the entire body). Each activity was completed once and one minute of free indoor walking was performed between them to facilitate their classification. The order of the activities was previously randomized. The Movemonitor classifies the activities into 5 categories (lying, sitting, standing, locomotion and shuffling) and the ActivPAL into 3 categories (sedentary, standing and stepping). Using the previously described algorithm, the data from the OPAL sensors were used to identify the beginning and end of each activity by detecting the walking phases that separated them.

**Table 4:** Summary of the activities performed during the activity recognition protocol.

Activity Type—Activity Recognition Protocol	Duration
**Standing**	2 min
**Taking the lift**	2 min
**Sitting and working at a computer**	2 min
**Lying**	2 min
**Ascending and descending steps**	1 min
**TOTAL**	**9 mins**

### Statistical analyses

Data analysis was performed using SPSS Statistics 21.0 (IBM Corporation, New York, USA).

For the investigation of step detection accuracy in the seven sensors, the mean absolute percentage error (MPE) for each sensor was computed as (1):

$$MPE=\frac{\left|\text{Ñ-N}\right|}{\text{N}}*100$$ (1)

The Kolmogorov-Smirnov test was used to analyse normality of data. As data were normally distributed, parametric tests were used and data were presented as mean and standard deviation (SD). Differences in group estimates between sensor outcomes were tested using mixed-model ANOVA with a significance level of p = 0.05 and post-hoc follow up analysis. Bland-Altman plots were used to assess the agreement between the measures and evaluate bias between the scores of the PAMs, where the difference (D) and the average (A) for each sensor were computed as:

D=Ñ−N (2)

$$M=\frac{Ñ+N}{2}$$ (3)

For the activity recognition protocol, the posture classifications given by the two monitors were extracted and expressed as a percentage of the duration as computed by the reference signals collected at the shanks.

## Results

The ANOVA showed significant differences in step count between the three walking speed conditions (p<0.05, see Table 3 for values). Planned contrasts revealed that the number of steps recorded at the self-selected speed was significantly lower than those at slow walking speed (p<0.01) and higher than those at fast walking speed (p<0.01).

**Fig 3:**
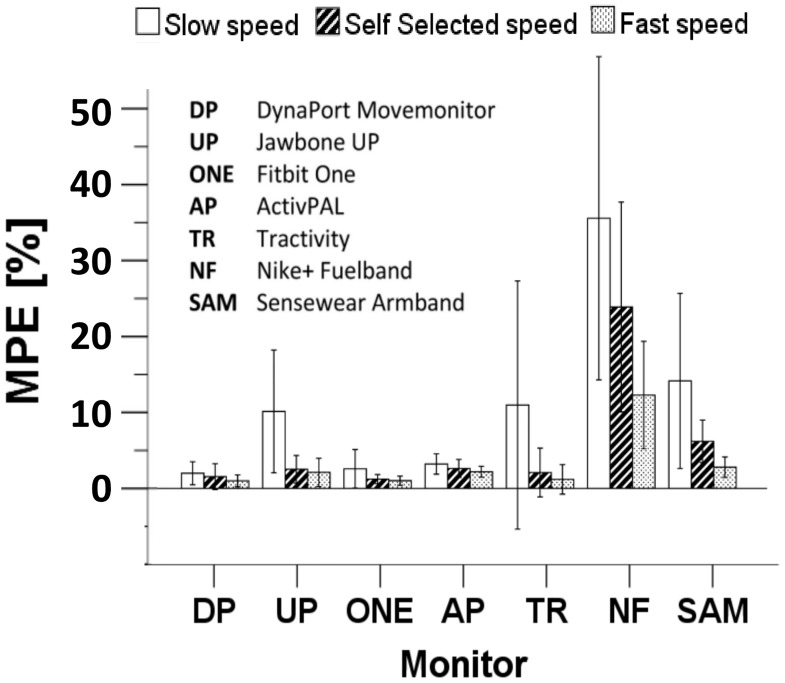
Summary of MPE for the 7 PAMs included in the study. The figure shows the mean percentage error (MPE) during slow, self-selected and fast walking speed trials for all the sensors included in the study. Error bars are mean ± SD.

There was a significant underestimation of Ñ for the Movemonitor, One, ActivPAL, Nike+ Fuelband and Sensewear Armband Mini, whereas the Tractivity significantly overestimated step count. The observed power was 0.999 for the overall ANOVA and ranged from 0.833 to 0.999 for the significantly different contrast tests. The Up did not show any systematic over- or underestimation. These findings were confirmed also when the data were separated by walking speed. Fig. 3 summarises mean and SD between individuals of the mean percentage error (MPE) at all walking speeds for each of the seven PAMs. The best performing device in terms of MPE was the Movemonitor, with MPE<2.0% at every speed, followed by One and ActivPAL, with MPE <2.6% and <3.2%, respectively. These three sensors presented also the smallest SD (≤1.7%, ≤2.5% and ≤1.5%, respectively). See the supporting information for the total number of steps (S1 Table) and MPE (S2 Table) for all the sensors included in the study.

**Fig 4:**
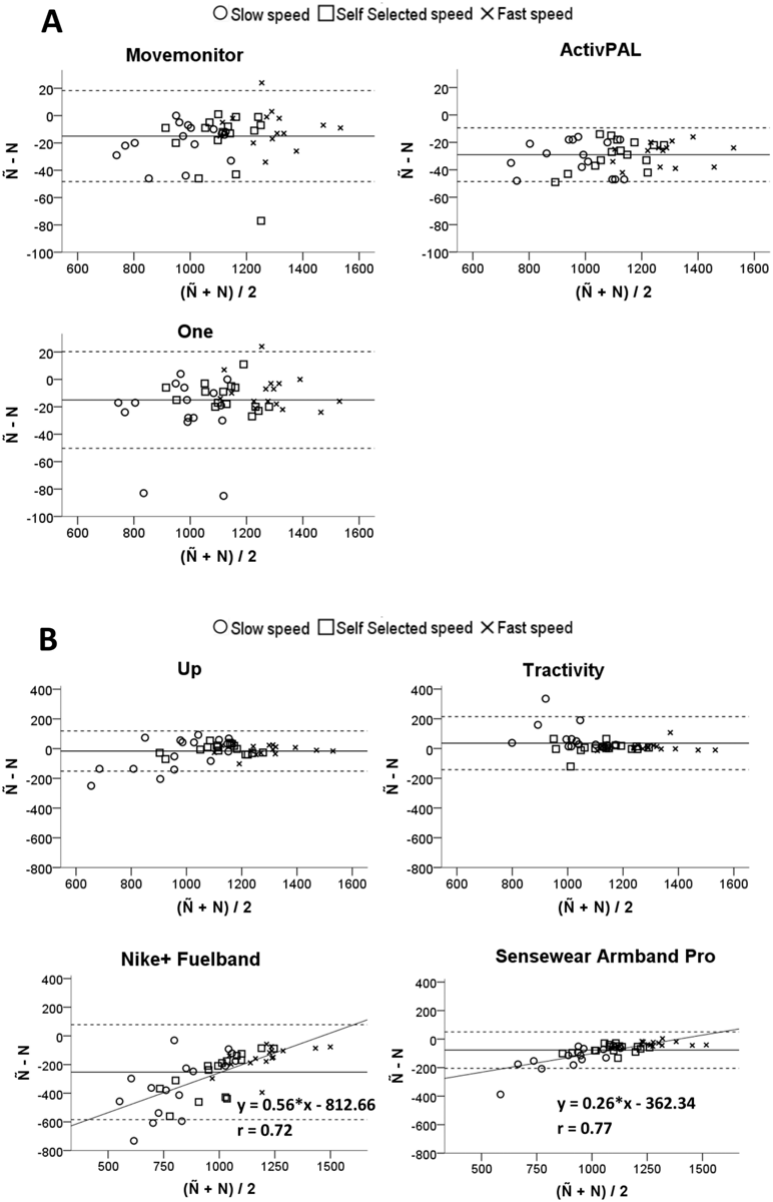
Bland-Altman plots for step count for (a) the Movemonitor, ActivPAL and One, and (b) for the Up, Tractivity, Nike+ Fuelband and Sensewear Armband Mini. The solid lines indicate the mean step count difference between the OPAL sensor and each monitor. The dashed lines indicate mean ± Limits of Agreement (1.96*SD). Regression lines, relevant equations and Pearson’s correlation coefficients (r) are shown for the Nike+ Fuelband and the Sensewear Armband Mini.

The Bland-Altman plots for the number of steps (Ñ), depicted in Fig. 4, showed an average ± Limits of Agreement (1.96*SD) underestimation of 15±33, 15±35,29±20, 16±135, 36±178, 253±331 and 77±127 steps for the Movemonitor, One, ActivPAL, Up, Tractivity, Nike+ Fuelband and Sensewear Armband Mini, respectively. The values of step count over- or underestimation for all the sensors at all walking speeds are shown in S3 Table of the supporting information. The correlation analysis (see regression lines on the Bland-Altman plots) highlighted also that for the Nike+ Fuelband and the Sensewear Armband Mini the underestimation was affected by the number of steps taken: the statistically significant (p<0.05) correlations between D and M were r = 0.72 and r = 0.77, respectively.

The results of the phase analysis performed on Movemonitor and ActivPAL data showed that, for both sensors, the best performance in terms of MPE was obtained during the outdoor walking: for the Movemonitor, MPE values ranged between 0.38±0.35% at natural walking speed and 0.54±0.65% at slow walking speed; for the ActivPAL, values ranged between 1.0±0.7% at fast walking speed and 1.4±0.8% at slow walking speed, respectively. The mixed-model ANOVA showed that the Movemonitor sensor was more accurate than the ActivPAL (p<0.05) in terms of MPE. The MPE also significantly differed in the 5 walking phases (p<0.001). There was also a significant interaction between speed and phase (p<0.01). Planned contrasts revealed that during the first transition phase (descending stairs), regardless of the sensor used, accuracy in step detection was higher during slow walking than at self-selected speed, while during the second transition phase (ascending stairs), MPE was lower at the self-selected speed than at slow walking speed, (p<0.05). Equally, accuracy in step detection was higher during the first transition phase at self-selected walking speed than at fast speed, while during the second transition phase, MPE was lower at the self-selected speed than at fast walking speed, (p<0.05).

The accuracy of the ActivPAL monitor in the classification of the activities performed during the activity recognition protocol (Table 5) ranged between 97.1% and 99.6%. Sitting while working at a computer was mainly categorized as sedentary activity (98.7%, excluding one outlier); taking the lift was mostly classified as standing (99.6% of the time). The accuracy of the Movemonitor device (Table 6) in classifying lying, sitting while working at a computer and stair walking ranged between 96.0 and 98.8%. Taking the lift was categorized either as standing or shuffling. Standing was categorized correctly only for 10.8% of the time, instead it was mainly classified as sitting (88.4%).

**Table 5:** Classification of the performed activities for the ActivPAL sensor.

	ActivPAL Categories		
Activity	Sedentary	Standing	Stepping
**Standing**	0.0 ± 0.0	**99.6 ± 0.8**	0.4 ± 0.8
**Taking the lift**	0.0 ± 0.0	**97.1 ± 3.2**	2.9 ± 3.2
**Sitting and working at a computer**	**98.7 ± 1.3**	0.7 ± 0.6	0.6 ± 1.0
**Lying**	**98.8 ± 1.6**	0.7 ± 0.9	0.5 ± 1.1
**Ascending and descending steps**	0.0 ± 0.0	1.4 ± 1.8	**98.6 ± 1.8**

Data is presented as percentage of the total duration of the activity (mean ± SD).

**Table 6:** Classification of the performed activities for the Movemonitor sensor.

	DynaPort Categories				
Activity	Standing	Sitting	Lying	Locomotion	Shuffling
**Standing**	10.8 ± 26.9	**88.4 ± 26.6**	0.0	0.7 ± 0.6	0.1 ±0.3
**Taking the lift**	**80.5 ± 5.8**	1.7 ± 6.5	0.0	3.0 ± 2.0	14.9 ± 3.5
**Sitting and working at a computer**	0.4 ± 0.5	**98.6 ± 1.4**	0.0	0.7 ± 0.8	0.3 ± 0.6
**Lying**	0.0	0.8 ± 1.1	**98.8 ± 1.4**	0.4 ± 0.6	0.0
**Ascending and descending steps**	0.7 ± 1.0	0.0	0.0	**99.2 ± 1.3**	0.1 ± 0.3

Data is presented as percentage of the total duration of the activity (mean ± SD).

## Discussion

It has been recently suggested that in activity monitoring research multiple comparison of monitors should be adopted to provide a better understanding of advantages or disadvantages of technology on the market [11]. The first aim of this study was to compare step counts of research and consumer-oriented PAMs during a short protocol including indoor and outdoor walking phases and stair climbing and descending. The second aim was to further characterise two of the chosen sensors in their ability to discriminate between simple and complex tasks and postures.

The experimental protocol adopted in this study proved to be suitable to investigate the accuracy of physical activity monitors. The chosen 11-minutes duration for the data collection allowed the highlighting of differences in the step count throughout the three walking speeds. Our experimental design did not include a quantitative measure of walking speed, which prevents us from making observations regarding the specific relationship between speed and accuracy of the PAMs. This could be of interest for applications involving patients or elderly individuals.

Five out of seven activity monitors underestimated the number of steps in all the three observed walking speeds (Movemonitor, One, ActivPAL, Nike+ Fuelband and Sensewear Armband Mini). The first three above-mentioned devices were also the three best performing in terms of MPE. For these three devices, no trend was found in the error, whereas for the latter two (Nike+ Fuelband and Sensewear Armband Mini), the underestimation was higher at the lower paces. This corroborates previous literature findings about the difficulty of step detection at slow walking speeds [15,16]. The reason for the poor performance of some PAMs is likely to be due to the fact that the products were originally developed for running. Also the Up accelerometer was markedly inaccurate at the lowest pace. The Tractivity was the only device that overestimated the steps at all walking speeds. The reason for this is not easily identifiable, since not enough information was available to the authors about the data processing techniques and algorithms, a problem also highlighted by Chen et al. [1].

The three best performing PAMs included the two devices explicitly designed for clinical use (Movemonitor and ActivPAL). These devices provide also the most complex activity reports including the classification of different activities such as lying, walking and standing. The One was the best consumer-based device in terms of MPE and might be the best low-cost option for step count monitoring.

When interpreting data measured from PAMs in real life conditions, careful consideration should be paid to the consequences of the bias existing between actual and measured steps. Since prolonged physical activity monitoring in clinical trials typically lasts up to one week [18], small underestimation of the time spent in an energy- and movement-demanding activity such as walking may be an amplifier for errors. For example, the one-week use of PAMs leading to underestimation errors higher than 14%, might translate into errors corresponding to ignoring more than one entire day of walking activity out of a seven-days observation period. Smaller errors, such as those found for the best performing monitors (1–3%), may be clinically irrelevant in the case of research studies involving sedentary populations, but might still need to be taken into account when investigating physical activity interventions. The phase analysis revealed that the best accuracy in step count was obtained during outdoor walking. This result might be explained by the fact that during indoor walking the likelihood of miscounting steps was higher than outdoor, since the participants had to stop-and-start to turn around at the end of the walkway, and the path they followed during free indoor walking was generally more tortuous than the one they walked outdoors. Nevertheless, the good performance of the sensors is encouraging for applications involving prolonged outdoor data collection.

The ActivPAL and Movemonitor performances in detecting steps were also examined in stair climbing during the two transition phases. Interestingly, for both sensors, at slow walking speed MPE was higher when ascending stairs than when descending. Conversely, at fast walking speed, MPE was higher when descending stairs than when ascending. At self-selected walking speed, the accuracy was not affected by whether the participant was ascending or descending stairs. This finding is in agreement with a previously reported study using pedometers [19] and should be the aim of further investigation to clarify what are the signal and software characteristics that might influence such an outcome.

The activity recognition protocol included all the activities indicated as recognisable by the manufacturers of the two tested sensors. Activities such as working at a computer or taking the lift were adopted to generate possible significant variations in the measured accelerations, so to include features entailing a realistic perturbation to the system. The results of this protocol showed that the position of the Movemonitor on the lower back of the participants leads to a high chance of misclassification of the standing posture, often confused with sitting. This problem, already highlighted in a previous validity study [20], is caused by the similar inclination of the accelerometers with respect to the gravity line during these two static activities [21]. Interestingly, despite the Movemonitor widely misclassified quiet standing, it correctly classified taking the lift as standing. Investigating the recognition capabilities of the Movemonitor during short activities (<5s), Dijkstra et al. [22] highlighted that short standing periods were well detected. Activity recognition methods employed in PAMs often rely on specific features in the signal to detect transitions between postures. Rapid and brief deceleration and acceleration of the lift may have helped the algorithm employed in the Movemonitor to correctly classify the standing posture during that specific task. Conversely, the location of the ActivPAL sensor on the thigh clearly overcomes the problem of static standing classification, but doesn’t allow separation of sitting from lying. Nevertheless, further studies should investigate the classification capabilities of these sensors in other groups such as older people or people with disability, to investigate how the activity recognition algorithms perform when pathologies hinder normal movement patterns.

PAMs are becoming increasingly available on the market and these devices are being used for research purposes in field-based applications and to promote population-wide physical activity. Within this framework, the information about the absolute error and variability of the output measures provided by this study could be used to model errors in PAMs’ data, in order to provide a better estimate of long-term physical activity, similarly to what was done by Nusser et al., who developed a measurement error model to match physical activity recall data based on questionnaires with an individual’s usual physical activity [23]. In addition, end-users aware of the inaccuracy of different PAMs might make better informed decision regarding the choice of the device to use for specific applications. We suggest that a similar approach to what has been done in this study, in which the reference step count is performed using protocols including the same tasks but for shorter periods than the ones used in this study, could be implemented as a spot check for patient specific calibration and reliability assessment of activity monitor devices, before giving them to patients for long-term monitoring.

## Conclusions

The overall step detection accuracy for the seven PAMs included in the study ranged between 0.9% (Movemonitor, fast walking speed) and 36.4% (Nike+ Fuelband, slow walking speed). On a practical point of view, considering a person taking 5000 steps per day (boundary between sedentary and low active lifestyle) over a one-week period this would translate into up to 326 and 12.737 steps not detected by the best and the worst performing PAM, respectively. The majority of the sensors underestimated the step count and Movemonitor, ActivPAL and One were the best performing PAMs in step count recognition. Movemonitor was the best performing device overall, but failed in the recognition of standing posture, usually misclassified as sitting. ActivPAL showed a good accuracy overall, although it is limited in not being able to discriminate between sitting and lying. One might be a valid low cost solution for monitoring the effect of interventions aiming at increasing the number of steps walked per day. Stair ascending and descending significantly affect step recognition accuracy, with a speed-dependent effect.

## Supporting Information

S1 Table Step count for the PAMs and the reference method. Values are mean ± SD.
(DOCX)

S2 Table Mean Absolute Percentage Error (MPE) for the PAMs. Values are mean ± SD.
(DOCX)

S3 Table Mean over- or underestimation of step count (D) for the PAMs. Values are mean ± SD.
(DOCX)
