# Supplementary material for: Step Detection and Activity Recognition Accuracy of Seven Physical Activity Monitors
Source: PLoS One. 2015 Mar 19;10(3):e0118723. doi: 10.1371/journal.pone.0118723 (PMC4366111; doi:10.1371/journal.pone.0118723)
Supplement: S1 Table — Values are mean ± SD. (DOCX) [file pone.0118723.s001.docx]

**Table S1. Step count for the PAMs and the reference method.** Values are mean ± SD.

| **Walking Speed** | **Duration** | **Movemonitor** | **Up** | **One** | **ActivPAL** | **Tractivity** | **Nike+ Fuelband** | **Sensewear Armband Mini** | **OPAL** |
| --- | --- | --- | --- | --- | --- | --- | --- | --- | --- |
| **Slow** | 11 min | 968 ± 131 | 952 ± 197 | 962 ± 128 | 955 ± 131 | 1081 ± 114 | 644 ± 246 | 858 ± 197 | 986 ± 127 |
| **Self selected** | 11 min | 1110 ± 100 | 1123 ± 107 | 1119 ± 103 | 1097 ± 111 | 1132 ± 108 | 865 ± 200 | 1059 ± 111 | 1127 ± 103 |
| **Fast** | 11 min | 1283 ± 117 | 1280 ± 117 | 1280 ± 112 | 1259 ± 120 | 1299 ± 117 | 1134 ± 159 | 1254 ± 119 | 1289 ± 115 |
