# Supplementary material for: Step Detection and Activity Recognition Accuracy of Seven Physical Activity Monitors
Source: PLoS One. 2015 Mar 19;10(3):e0118723. doi: 10.1371/journal.pone.0118723 (PMC4366111; doi:10.1371/journal.pone.0118723)
Supplement: S2 Table — Values are mean ± SD. (DOCX) [file pone.0118723.s002.docx]

**Table S2. Mean Absolute Percentage Error (MPE) for the PAMs.** Values are mean ± SD.

| **Walking Speed** | **Movemonitor** | **Up** | **One** | **ActivPAL** | **Tractivity** | **Nike+ Fuelband** | **Sensewear Armband Mini** |
| --- | --- | --- | --- | --- | --- | --- | --- |
| **Slow** | 1.98 ± 1.50 | 10.08 ± 8.04 | 2.56 ± 2.53 | 2.99 ± 1.51 | 10.92 ± 16.26 | 35.39 ± 21.17 | 14.08 ± 11.47 |
| **Self selected** | 1.54 ± 1.69 | 2.51 ± 1.80 | 1.13 ±0 .65 | 2.45 ± 1.31 | 2.07 ± 3.20 | 23.76 ± 13.75 | 6.16 ± 2.79 |
| **Fast** | 0.93 ±0.79 | 2.10 ± 1.85 | 1.01 ±0 .59 | 2.04 ± .88 | 1.17 ± 1.94 | 12.22 ± 7.04 | 2.77 ± 1.34 |
