# Supplementary material for: Step Detection and Activity Recognition Accuracy of Seven Physical Activity Monitors
Source: PLoS One. 2015 Mar 19;10(3):e0118723. doi: 10.1371/journal.pone.0118723 (PMC4366111; doi:10.1371/journal.pone.0118723)
Supplement: S3 Table — Values are mean ± SD. (DOCX) [file pone.0118723.s003.docx]

**Table S3. Mean over- or underestimation of step count (D) for the PAMs.** Values are mean ± SD.

| **Walking Speed** | **Movemonitor** | **Up** | **One** | **ActivPAL** | **Tractivity** | **Nike+ Fuelband** | **Sensewear Armband Mini** |
| --- | --- | --- | --- | --- | --- | --- | --- |
| **Slow** | -19 ± 13 | -35 ± 110 | -25 ± 26 | -31 ± 12 | 95 ± 134 | -343 ± 204 | -129 ± 86 |
| **Self selected** | -17 ± 21 | -4 ± 34 | -12 ± 10 | -29 ± 11 | 4 ± 40 | -262 ± 147 | -68 ± 28 |
| **Fast** | -9 ± 14 | -9 ± 34 | -9 ± 12 | -28 ± 8 | 10 ± 28 | -155 ± 88 | -35 ± 17 |
| **Overall** | -15 ± 17 | -16 ± 69 | -15 ± 18 | -29 ± 10 | 39 ± 91 | -253 ± 169 | -77 ± 65 |
